# Undifferentiated pleomorphic sarcoma of the lateral thigh with KRAS/NF1 co-mutation recurred after repetitive surgical resection: A case report and review of the literature

**DOI:** 10.3389/fsurg.2022.842054

**Published:** 2022-10-21

**Authors:** Shuo Zhang, Zimo Zhou, Jing Xu

**Affiliations:** ^1^ Department of Plastic Surgery, The First Affiliated Hospital of Bengbu Medical College, Bengbu, China; ^2^ Department of Orthopedics, Shengjing Hospital of China Medical University, Shenyang, China

**Keywords:** undifferentiated pleomorphic sarcoma, recurrence, KRAS, NF1, case report

## Abstract

Undifferentiated pleomorphic sarcoma (UPS), a rare soft tissue sarcoma subtype, mainly occurs in the deep parts of the limbs and trunk, observed as rapidly growing painless lumps, rarely located under the skin or protrudes from the skin surface. The risk of recurrence and metastasis is associated with multiple factors. Mutation of tumor gene, tumor occurrence, location and depth of invasion, and tumor size have great influence on prognosis. In this study, we described a case of UPS with KRAS/NF1 co-mutation. This case had undergone UPS extended resection for four times combined with chemotherapy in another hospital. The resection area was more than 3 cm, and tumor relapsed after all operations. This time, the tumor protruded from the left lateral surface with ulceration and infection. Due to multiple surgeries, the anatomy of the lateral femoral vessels has been seriously damaged. We performed expanded tumor resection and adjacent flap transfer repair; meanwhile, vacuum sealing drainage (VSD)-negative pressure closed the drainage, and the patient recovered well after surgery. After surgery, the patient was transferred to the Department of Oncology for chemotherapy. There was no recurrence after 6 months of follow-up. Gene mutation plays an important role in UPS recurrence and metastasis. At the same time, occurrence and location, depth, and size of UPS have great influence on the prognosis. Deeper exploration and expanded resection are important for the prognosis of UPS.

## Introduction

Undifferentiated pleomorphic sarcoma (UPS), as a type of skin and soft tissue tumors, is a malignancy of mesenchymal origin (1). UPS most commonly involves the limbs, and it was also reported to be metastatic to head and neck (2). In the past, UPS has been difficult to distinguish from other mesenchymal tumors such as dermal sarcomas; with the development of histologic diagnosis, immunohistochemical methods can make it easier to rule out other tumor types and make a definitive diagnosis (3). UPS has a high recurrence rate and becomes more aggressive with the depth of occurrence and invasion, especially infiltrating into fat or deeper tissue (1, 4, 5). In the past, Ben-Izhak and Groisman (6) first reported a case of soft tissue malignant fibrous histiocytoma with mutation of neurofibromin 1 gene (NF-1). Subsequently, some reports indicated that mutation of NF-1 gene can affect the UPS (7, 8). Another gene mutation, KRAS, was first reported in UPS by Li et al. (9). KRAS is frequently mutated in tumors and may promote tumor development. In this article, we reported a case of recurrent undifferentiated pleomorphic sarcoma after five times of excision. Co-mutation of KRAS and NF-1 may play an important role in this case. At present, clinicians have little understanding of this tumor, and the initial diagnosis is difficult. The clinical manifestations, diagnosis, and treatment of this disease have not been established yet. Here, we reported a case of recurrent UPS after five resections, including clinical manifestations, pathological findings, genetic testing, and treatment options.

## Case report

**Figure 1:**
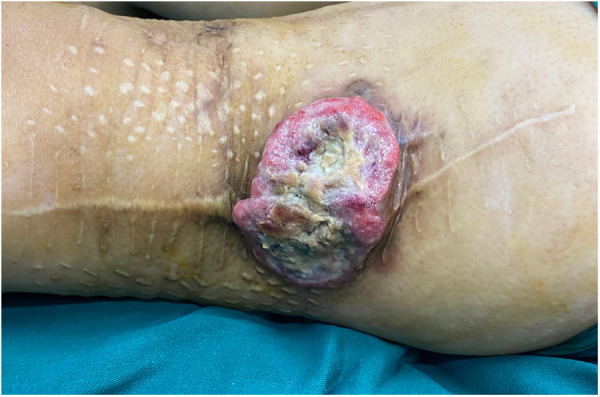
The tumor that has protruded from the surface is accompanied by ulceration.

A 61-year-old Asian female presented with a 2-month history of a recurrent mass with no pain, and the mass had broken through the skin and had ulcerated. This patient first found the mass on the lateral thigh 8 years ago. The initial diameter was about 1.0 cm, and it grew to 5 cm in 3 months, protruding from the skin surface with slight pain. The patient was admitted to the local hospital and underwent extended tumor resection. Postoperative pathology showed that the resection margin and basal resection margin were negative. Postoperative immunohistochemical examination, Vimentin (+), Ki-67 (+, 30%), BCL2 (+), CD31 (−), CD34 (−), and S100 (−), combined with pathology were consistent with the diagnosis of UPS. Within 7 years after this operation, the tumor was resected again for three times, all of which were extended to the muscle layer. Postoperative pathology showed that all the surgical margins were negative and consistent with the diagnosis. The patient received chemotherapy after all four operations. This time, physical examination revealed a tumor (8 cm × 9 cm), which has partially protruded out from surface (5 cm × 3 cm) with ulceration (Figure 1).

CT results demonstrated there is a large soft tissue mass in postoperative anterolateral area of the left mid-thigh, involving the superficial. No significant enlargement of abnormal lymph nodes was observed in the abdominal cavity and groin. This patient was contraindicated for MR because of with cardiac stenting. Previous pathologic findings of the tumor suggested spindle cell tumor. Combined with clinical manifestations and immunohistochemical results, this case was diagnosed as UPS.

Bacterial culture of purulent pus at the site of tumor ulceration revealed infection of *Providencia rettgeri*. Drug sensitivity test results suggested that *P. rettgeri* was resistant to cotrimoxazole, ampicillin, and cefazolin. Additional CT workup revealed multiple two-sided pulmonary nodules that were suspicious for metastatic disease.

**Figure 2:**
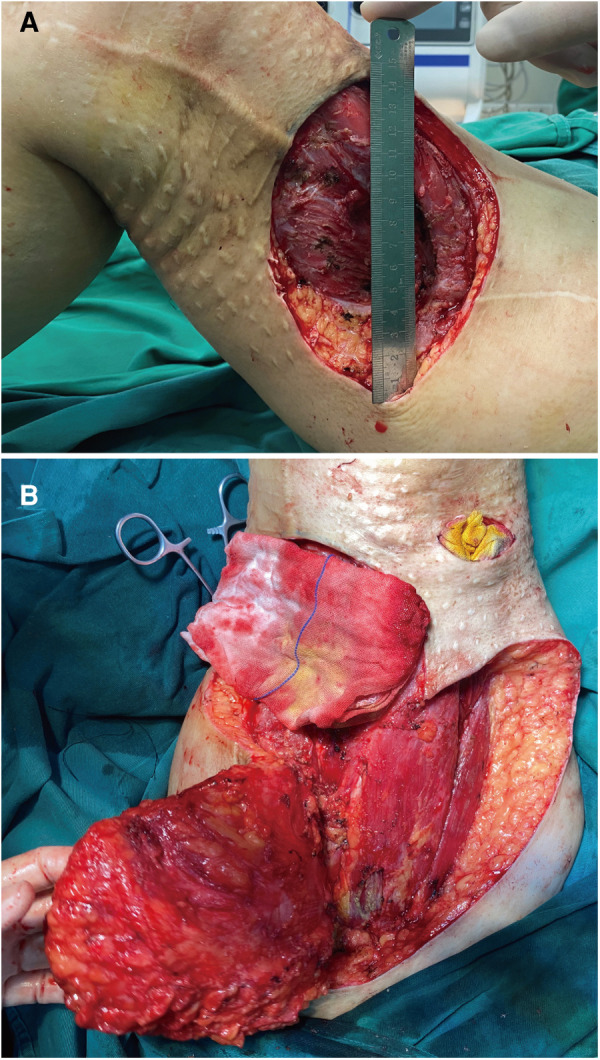
Intraoperative image. (**A**) Appearance after surgical resection of UPS. (**B**) Image showing the muscle and adipose tissues exposed after expanded tumor resection.

Considering the patient's previous tumor recurrence and personal wishes, we performed surgery after antibiotic therapy for *P. rettgeri*. We performed vascular examination of the medial and medial thigh using Doppler ultrasound and found abundant blood supply here. Therefore, the treatment of free flap and direct skin grafting were excluded. The anteromedial thigh adjacent flap was used for repair. In the resection of the tumor, a double layer of gauze was used to suture the part to be removed, and the replanting of tumor cells during the resection was avoided by covering the tumor. The diameter of operative incision was 15 cm (Figure 2A). Intraoperative exploration revealed that the infiltration depth of the tumor had reached the vastus lateralis muscle, and the muscle layer at this depth had been damaged, which may be related to the previous operation. We did a complete removal from the basilar part of the tumor. During surgical resection, we performed more adequate tissue exposure and extended resection, especially potentially overlooked tumor tissue in deeper muscle tissue were the focus of intraoperative attention. Intraoperative exploration of lymph nodes, lymphatics, and nerves revealed no invasion of nerves or lymphoid tissue. In the end, we performed adjacent flap transfer repair; meanwhile, vacuum sealing drainage (VSD) negative pressure closed the drainage (Figure 2B).

**Figure 3:**
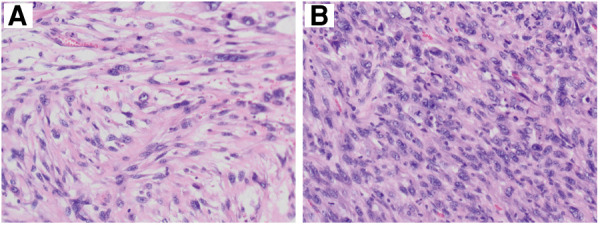
Histopathological features of tumor. Microscopically, numerous spindle cells are seen (×200). **A** and **B** mean the tissue sections of different parts in the mass.

**Figure 4:**
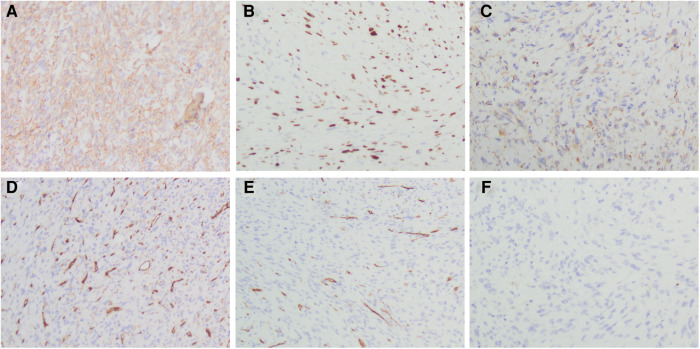
Immunohistochemistry staining of differentially expressed proteins in the tumor tissue. (**A**) Tumor cells showing positive immunoreactivity for SMA. (**B**) Tumor cells showing positive immunoreactivity for Ki-67 (index 70%). (**C**) Tumor cells showing positive immunoreactivity for Desmin. (**D**) Tumor cells showing no immunoreactivity for CD31 (×200). (**E**) Tumor cells showing no immunoreactivity for CD34 (×200). (**F**) Tumor cells showing no immunoreactivity for S100 (×200).

Histopathological section showed that there are numerous spindle cells with obvious atypia and irregular nuclei; the mitotic figures were extremely common to see, and multinucleated giant cells are seen, which is consistent with UPS (Figure 3). The tumor cells were immunohistochemically determined to be positive for SMA, Ki-67 (index 70%), and Desmin but negative for CD31, CD34, and S100 (Figure 4).

The Genetic test on UPS of this case suggested that KRAS mutation (NM_033360: exon2: c. G38A: p.G13D G13D; Mutation abundance: 0.87%) and NF1 mutation (NM_000267: exon36: c.5057_5079del: p. V1686fs; Mutation abundance: 57.03%).

Considering the multiple pulmonary nodules and the high risk of UPS recurrence, this case was transferred to the Department of Chemotherapy for combination chemotherapy with cyclophosphamide, and doxorubicin. The patient’s survival is good. There was no recurrence after 6 months of follow-up. A written informed consent was obtained from the patient.

## Discussion

UPS is a rare spindle cell tumor that originates in the skin or soft tissue. In the past, it was identified differently. When the tumor springs in different tissues, it can be called as atypical fibroxanthoma, superficial UPS, or pleomorphic dermal sarcoma and deep UPS (1). Though immunohistochemistry, it can be differentiated more precisely and easily. The depth of UPS invasion often is an important indicator of the prognosis of advanced tumors, though histopathological examination can be used to determine the aggressiveness of the tumor to a certain degree (1, 10). One study showed that when the tumor infiltrates deeper tissue, the recurrence rate and malignancy degree are higher (1), which is consistent with the characteristics of this case. However, the relationship between tumor depth and mortality was insignificant. In reviewing relevant case reports and recent studies, we found that the prognosis and survival of UPS patients with deep tissue were different, which may also be related to the low sample size (11–16).

Ras gene is a kind of oncogene, which has been widely concerned, and its mutation provides an important direction for tumor targeted therapy. KRAS mutation as the most common type of RAS, and it occurs in 30% of patients with soft tissue (17). Li et al. (9) reported a 66-year-old female UPS patient with KRAS mutation who died 1 year after surgery due to metastasis and recurrence, which may prompt that KRAS mutation plays a key role in UPS recurrence.

NF1 was classified as a tumor suppressor because of its loss of function in neurofibromatosis Type I, and Neurofibromatosis Type I patients were more susceptible to other malignancies (18). Interestingly, one study showed the relationship between NF1 and RAS/MAPK signaling pathway (19). In this patient, NF1/KRAS co-mutation may be an important factor in repeated recurrence and metastasis of UPS. However, there is still a lack of more evidence to prove that genetic mutations are directly related to the recurrence of UPS. Therefore, some basic studies on the occurrence and development of UPS are necessary.

Furthermore, as one of the treatment methods for UPS, surgical treatment is often the first choice. There are more attentions should be paid to the following aspects:

(1) We noted that in some case reports, the excision of the tumor and area of tumor margins were not described in great detail (13–15). In such patients, more attention should be paid to the depth and size of tumor invasion during surgery, especially during surgical resection, more adequate tissue exposure and expanded resection are necessary, and we need to explore deeper tissue to find tumor tissue that might have been overlooked.
(2) The main supply vessels in the lateral femoral region of the thigh included the descending branch of the lateral circumflex femoral artery, multiple intermuscular perforators, or musculocutaneous perforators. For patients without previous operation history, free flaps can be used to repair the defect. However, after the anatomical structure of the muscle was damaged, in the tumor resection or the repair of flap, we should always pay attention to ensure whether the blood vessels were injured or ligation during the previous operation, in order to avoid secondary injury during the operation. The pedicle was partially detached for the vessels that had been ligated or needed to be ligated before, so that the pedicle was retained with sufficient length and marked with silk thread. In addition, it was not necessary to remove the blood vessels that have been ligated in previous surgeries, so as to avoid bleeding that could not be handled due to the shortening of the blood vessel after detachment. For the sake of safety, it is the best choice to select the anteromedial thigh adjacent flap for repair.
(3) In addition, after extensive resection of tumor, wound repair is very important. In the past, free flaps such as rectus abdominis and rectus femoris can be used to repair defects in the muscle layer on the lateral thigh. However, this patient had undergone multiple surgeries, and the structure had been destroyed; the diameter of the remaining wound can often reach 15 cm. Therefore, it is very important to design flaps according to the size of the wound. The ratio of the length and width of the flaps should not exceed 1.5:1, and gauze was used to simulate the flap for transfer to avoid the occurrence of too small flaps during the operation.
(4) When obtaining the flap, a sharp blade can be used to cut the skin layer and the fat layer. When getting below the fat layer, the surgeon should avoid using sharp instruments, such as blades and electric knives, to prevent damaging blood vessels and nerves. After a retracting hook is used to fully expose the bottom layer of the flap, a vascular clamp can be used to carefully peel off the flap, paying attention to protecting the vascular pedicle. When finding the vascular pedicle supplying the flap, we did not dissect it much. In addition, a large amount of fascia and fat layer were preserved to facilitate the venous return of the flap. When the transfer angle and distance are sufficient, further separation of the flap should be stopped to avoid blood supply disturbance. Finally, the donor site of the flap was repaired by one-stage skin grafting combined with VSD.

## Conclusion

In conclusion, our identification of a point co-mutation in KRAS and NF1 in UPS suggests the possibility that point mutation of this oncogene may be important in the recurrence and metastasis. Moreover, the location of UPS, depth, and size of invasion are related to UPS metastasis and recurrence. It is very important for the surgeon to explore the tumor and surrounding tissues during the operation, and further exploration and expanded resection may have a more positive effect on the prognosis of the tumor. In tumor resections after UPS recurrence, surgeons need to consider the destruction of the existing anatomical structure and vascular injury. During the operation, it is necessary to protect the remaining healthy tissue as much as possible and adopt a reasonable flap transplantation method to ensure the recovery of the patient.

## Data Availability

The original contributions presented in the study are included in the article/Supplementary Material, further inquiries can be directed to the corresponding author.
